# Feasibility and repeatability of localized ^31^P‐MRS four‐angle saturation transfer (FAST) of the human gastrocnemius muscle using a surface coil at 7 T

**DOI:** 10.1002/nbm.3445

**Published:** 2015-12-18

**Authors:** Marjeta Tušek Jelenc, Marek Chmelík, Wolfgang Bogner, Martin Krššák, Siegfried Trattnig, Ladislav Valkovič

**Affiliations:** ^1^ High Field MR Centre, Department of Biomedical Imaging and Image‐Guided TherapyMedical University of ViennaViennaAustria; ^2^ Christian Doppler Laboratory for Clinical Molecular MR ImagingViennaAustria; ^3^ Division of Endocrinology and MetabolismDepartment of Internal Medicine IIIMedical University of ViennaViennaAustria; ^4^ Department of Imaging MethodsInstitute of Measurement ScienceSlovak Academy of SciencesBratislavaSlovakia; ^5^ Oxford Centre for Clinical MR Research (OCMR)University of OxfordUnited Kingdom

**Keywords:** ^31^P‐MRS, saturation transfer, FAST, 7 T, muscle metabolism

## Abstract

Phosphorus (^31^P) MRS, combined with saturation transfer (ST), provides non‐invasive insight into muscle energy metabolism. However, even at 7 T, the standard ST method with *T*_1_^app^ measured by inversion recovery takes about 10 min, making it impractical for dynamic examinations. An alternative method, i.e. four‐angle saturation transfer (FAST), can shorten the examination time. The aim of this study was to test the feasibility, repeatability, and possible time resolution of the localized FAST technique measurement on an ultra‐high‐field MR system, to accelerate the measurement of both P_i_‐to‐ATP and PCr‐to‐ATP reaction rates in the human gastrocnemius muscle and to test the feasibility of using the FAST method for dynamic measurements.

We measured the exchange rates and metabolic fluxes in the gastrocnemius muscle of eight healthy subjects at 7 T with the depth‐resolved surface coil MRS (DRESS)‐localized FAST method. For comparison, a standard ST localized method was also used. The measurement time for the localized FAST experiment was 3.5 min compared with the 10 min for the standard localized ST experiment. In addition, in five healthy volunteers, P_i_‐to‐ATP and PCr‐to‐ATP metabolic fluxes were measured in the gastrocnemius muscle at rest and during plantar flexion by the DRESS‐localized FAST method.

The repeatability of PCr‐to‐ATP and P_i_‐to‐ATP exchange rate constants, determined by the slab‐selective localized FAST method at 7 T, is high, as the coefficients of variation remained below 20%, and the results of the exchange rates measured with the FAST method are comparable to those measured with standard ST.

During physical activity, the PCr‐to‐ATP metabolic flux decreased (from *F*_CK_ = 8.21 ± 1.15 mM s^−1^ to *F*_CK_ = 3.86 ± 1.38 mM s^−1^) and the P_i_‐to‐ATP flux increased (from *F*_ATP_ = 0.43 ± 0.14 mM s^−1^ to *F*_ATP_ = 0.74 ± 0.13 mM s^−1^).

In conclusion, we could demonstrate that measurements in the gastrocnemius muscle are feasible at rest and are short enough to be used during exercise with the DRESS‐localized FAST method at 7 T. © 2015 The Authors. *NMR in Biomedicine* published by John Wiley & Sons Ltd.

**Abbreviations used**
AMARES: Advanced Method for Accurate, Robust, and Efficient Spectral Fitting
BIR4: adiabatic plane rotation pulse
BMI: body mass index
CK: creatine kinase
CV: coefficient of variation
DRESS: depth‐resolved surface coil MRS
F: metabolic flux
FA: flip angle
FAST: four‐angle saturation transfer
IR: inversion recovery
ISIS: image selected in vivo spectroscopy
k: exchange rate constant
NA: number of averages
PCr: phosphocreatine
P_i_: inorganic phosphate
SD: standard deviation
SAR: specific absorption rate
ST: saturation transfer
T_1_^app^: apparent longitudinal relaxation
TRiST: triple repetition time saturation transfer.

## Introduction

Phosphorus (^31^P) MRS provides non‐invasive insight into muscle energy metabolism. Metabolic alterations are related to several diseases, e.g. heart failure, stroke, and muscle disease. Therefore, ^31^P‐MRS has become the standard method for studying metabolic processes *in vivo* 1, 2, 3, 4, 5.

^31^P‐MRS with frequency selective saturation transfer (ST) can be used to study reaction rates and metabolic fluxes 6, 7. The general principle of ST measurements is to “magnetically label” the phosphorous nuclei in one molecule and monitor how fast this molecule chemically transforms into another 8. Thus, ^31^P MRS‐ST methods offer the ability to examine the key bioenergetic reactions of creatine kinase (CK) (ATP ⇆ phosphocreatine (PCr) exchange), which is abundant in skeletal muscle 9, 10, and the ATP synthesis/hydrolysis cycle (ATP ⇆ inorganic phosphate (P_i_) exchange). Alterations of these have been associated with insulin resistance, type 2 diabetes, and aging 10, 11, 12.

An ST experiment is an excellent alternative method with which to study the energy metabolism of organs that cannot be challenged by exercise in the MR scanner (e.g. the brain 13 or the liver 14, 15), and is, therefore, also suitable in muscle studies for immobilized and/or uncooperative patients 16, 17.

However, the current measurement time restricts the application of ST techniques in time‐resolved experiments. At clinical field strength (≤3 T), the whole standard ST experiment, consisting of transfer and control experiments and inversion recovery (IR) based *T*_1_^app^ measurements, requires 30–80 min 17, 18, 19, 20, and, even at ultra‐high field (7 T) strength, the measurement of reaction rates with a standard ST measurement requires 10 min 21. This is still too long for application in dynamic studies of exchange rates (*k*), or in pathologies that affect muscle metabolic homeostasis. In particular, the possibility to measure CK equilibrium and ATP turnover in skeletal muscle during ischemia, euglycemic–hyperinsulinemic clamps, and/or dynamic exercise would be of high interest.

Using a rapid alternative method, called four‐angle saturation transfer (FAST), presented by Bottomley *et al.* for the measurement of the CK reaction rate at 1.5 T 18, it might be possible to shorten the examination time. The FAST method avoids the time consuming IR based *T*_1_^app^ experiment and replaces it with the efficient dual‐angle method 18.

The accuracy of FAST relies on the accuracy of the excitation flip angles (FAs) used for the dual‐angle‐based *T*_1_ measurements. The FAST technique was originally described at 1.5 T using 4 ms long adiabatic *B*_1_‐insensitive rotation (BIR4) excitation pulses, which ensure accurate FAs without the need for calibration before each experiment, even in the presence of strong *B*_1_^+^ inhomogeneities 18. Therefore, adiabatic BIR4 excitation pulses would also be preferable to conventional pulses (e.g. sinc) for the FAST technique at 7 T. However, the power requirements to fulfill adiabatic condition and specific absorption rate (SAR) limitations, in particular in combination with saturation pulses, hinder their applicability at higher magnetic fields (>1.5 T) 22, 23.

Alternatively, conventional pulses that allow spatial localization, i.e. used in a depth‐resolved surface coil MRS (DRESS) sequence 24, may be used, but require accurate pulse calibration. DRESS employs a slice selective excitation in conjunction with surface coil detection to localize NMR signals to a plane of preselected thickness and location 24.

The aim of this study was to test the feasibility, repeatability, and possible increase in temporal resolution of DRESS‐localized FAST measurements at 7 T, to accelerate the measurement of both P_i_‐to‐ATP (ATP synthesis) and PCr‐to‐ATP (CK) reaction rates in the human gastrocnemius muscle. The unidirectional exchange rate constants (*k*), measured by FAST at rest, were compared with the exchange rate constants measured with standard ST at 7 T. Finally, the feasibility of using the FAST method for dynamic experiments was tested, to determine the PCr‐to‐ATP and P_i_‐to‐ATP exchange rates and fluxes in the exercising gastrocnemius medialis muscle at 7 T.

## Experimental Details

All MR examinations were performed on a 7 T MAGNETOM MR system (Siemens Healthcare, Erlangen, Germany) equipped with a double‐tuned (^31^P/^1^H), circular surface coil of 10 cm diameter (Rapid Biomedical, Rimpar, Germany). The maximum available gradient strength of the used MR system is 40 mT m^−1^ per direction.

### Phantom experiments

To test conventional sinc‐shaped pulses for excitation, a 1000 ml large, glass, cylindrical shaped phantom filled with solution containing 20 mM P_i_ was used. For this purpose, the signal intensity of P_i_ was assessed over a range of excitation frequency offsets (i.e. −20 ppm to 20 ppm in steps of 0.5 ppm). The pulse profile of a conventional excitation pulse (i.e. sinc pulse duration 600 µs, for FA = 15° and FA = 52°) was measured and was used for excitation.

Due to the *B*_1_^+^ inhomogeneity of the single‐loop surface coil, its utility for homogeneous FA excitation is limited. However, its application in a defined volume, i.e. a narrow slab parallel or almost parallel to the coil, selected using a DRESS localization sequence, might provide a feasible alternative.

To evaluate the FA distribution of the sinc excitation pulse (with 600 µs duration), *B*_1_^+^ maps were measured in the same phantom using the surface coil. The FA map was calculated based on the acquisition of two steady‐state ^31^P‐MRSI experiments with two different *T*_R_ values 25. The selection of *T*_R_ values depends on the *T*_1_ of the phantom (*T*_1_ = 2.3 s, which was measured by an IR experiment), and on the range of expected FAs, which determines the *T*_R long_/*T*_R short_ ratio. Thus, the *T*_R_ values used were *T*_R long_ = 2.76 s and *T*_R short_ = 0.46 s. Other parameters were set as follows: field of view = 20 × 20 cm^2^; number of averages (NA) = 1; *T*_E_* = 0.7 ms; 32 × 32 voxels; eight prep scans.

*B*_1_‐field maps for both nominal FAs, 15° and 52°, were acquired, and the off‐resonance behavior was investigated by changing the excitation offsets (±20 ppm) of the sinc pulse.

The FA distribution was then also measured *in vivo* in five volunteers using the same method as described above, but with the matrix size of 16 × 16 voxels.

### In vivo measurements

**Figure 1:**
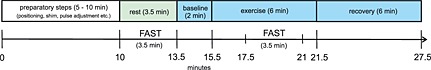
Schematic representation of the measurement protocol. The measurement preparation was followed by the FAST measurement at rest and by the dynamic experiment. Each dynamic experiment consisted of the acquisition of baseline data during 2 min of rest, 6 min of aerobic plantar flexion exercise (including FAST measurement), and 6 min of recovery.

The feasibility of the FAST method *in vivo* at 7 T was tested in eight healthy, lean volunteers (two female/six male; mean age 28.8 ± 2.8 years, body mass index (BMI) = 22.1 ± 2.5 kg m^−2^). All participants were lying in the supine position, with the right calf muscle placed on top of the coil. For dynamic experiments, five healthy volunteers (two female/three male; mean age 28.4 ± 1.6 years, BMI = 23.5 ± 2.1 kg m^-2^) were also investigated, lying in the supine position on an ergometer dedicated for plantar flexion exercise (Trispect, Ergospect, Innsbruck, Austria). The study protocol (Fig. 1) was approved by the local ethics committee, and written, informed consent was obtained from all volunteers.

### FAST *in vivo* examination

Based on the phantom experiments, a sinc excitation pulse (duration 600 µs, excitation bandwidth 6.4 kHz, chemical shift displacement error (CSDE) ±0.14 cm for ±600 Hz difference between γ‐ATP and P_i_) was used for *in vivo* DRESS‐localized FAST examinations at 7 T.

**Figure 2:**
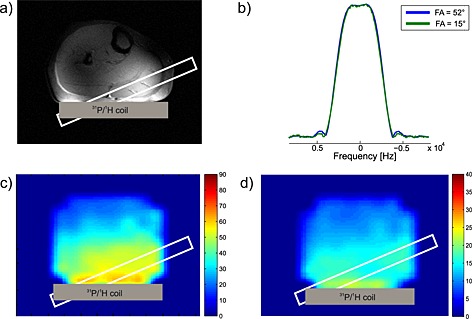
(a) Localizer image of a calf muscle with depicted slice selection of the DRESS localization sequence. The thickness of the slab represents the chemical shift displacement between PCr and P_i_. (b) Excitation sinc pulse profiles for 15° (green line) and for 52° (blue line) FA. The profiles were scaled to their maximum. (c, d) Maps of FA distribution of excitation SINC pulse used with nominal FA = 52° (c) and FA = 15° (d) measured in a volunteer.

After acquisition of the localizer images, a 15 mm thick MRS selection slab representing the volume of interest was placed in the medial gastrocnemius muscle in each volunteer individually based on a localizer image. An example of the slab positioning in the medial gastrocnemius muscle is shown in Fig. 2a. RF power was adjusted for the maximum of localized PCr signal, by varying the RF transmit voltage, and *B*_0_ shimming was performed to optimize the magnetic field homogeneity. For the assessment of metabolite concentrations, the initial acquisition without saturation (FA = 90°, *T*_E_* = 0.4 ms, *T*_R_ = 15 s, NA = 16) was recorded at rest, prior to the FAST experiment, and the γ‐ATP was used as an internal reference.

To determine *T*_1_^app^, DRESS‐localized FAST measurements were made in two experiments, the first with a nominal FA of 52° and NA = 8, and the second with a nominal FA of 15° and NA = 24. Both experiments were performed with γ‐ATP saturation (frequency offset of −2.48 ppm), with the control experiment for the PCr‐to‐ATP exchange rate at a downfield frequency mirrored around PCr at 2.48 ppm, and with the control experiment for the P_i_‐to‐ATP exchange rate at a downfield frequency mirrored around the P_i_ resonance at 12.52 ppm. *T*_R_ = 2 s, *T*_E_* = 0.4 ms, and four preparation scans were used. To test the repeatability of the measurement protocol, the FAST experiment was repeated four times within one session in six of the recruited volunteers.

For comparison and validation of the proposed method, the reaction rates were also measured using the DRESS‐localized standard ST method, with *T*_R_ = 15 s, *T*_E_* = 0.4 ms, NA = 8, and FA = 90°. *T*_1_^app^ was measured in an IR experiment during continuous irradiation of the γ‐ATP resonance (eight *T*_I_ values of 100, 300, 500, 700, 1500, 3000, 5000, and 8000 ms; NA = 2) on the identical volume of interest.

The measurement time for the localized FAST experiment was 3.5 min compared with the 10 min required for the localized standard ST experiment.

### Dynamic FAST *in vivo* examination

Measurements during exercise were made using the same protocol for DRESS‐localized FAST measurements as described above. First the complete FAST experiment was performed at rest, and second the FAST experiment with the same parameters was performed during plantar flexion (one flexion per *T*_R_) exercise, at the work load of approximately 20% of maximal voluntary contraction force. FAST measurements during exercise started 2 min after the onset of exercise, when the steady state of PCr depletion is typically reached 26, 27 (Fig. 1).

### Data processing, calculations, and statistical analysis

All measured spectra were analyzed using jMRUI (Java‐Based Magnetic Resonance User Interface; version 5) with the AMARES (Advanced Method for Accurate, Robust, and Efficient Spectral Fitting) time‐domain fitting algorithm 28. PCr and P_i_ resonance lines were fitted as single Lorentzians, whereas γ‐ and α‐ATP signals were fitted as Lorentzian doublets of equal amplitude separated by 16 Hz and β‐ATP was modeled as a triplet with the central peak double the amplitude of the two side peaks separated by ±16 Hz. To fit the spectra obtained during the saturation IR experiment, the zeroth‐ and first‐order phase and linewidths of PCr and P_i_ signals were determined from the last IR scan (i.e. with *T*_I_ = 8000 ms). This provides sufficient stability to fit IR spectra with peaks of different *T*_I_ values using the AMARES algorithm 21. For the FAST experiment the linewidths of PCr and P_i_ were determined from the highest‐signal‐to‐noise‐ratio spectra and then constrained to ±5 Hz.

For the standard ST experiment, the apparent relaxation times, *T*_1_^app^, obtained during continuously irradiating γ‐ATP resonance, were calculated in MATLAB (MathWorks, Natick, MA, USA), based on a three‐parameter least‐squares fit to the varying *T*_I_ data.

For the FAST experiment, both fully relaxed magnetizations of PCr and P_i_ (*M*_0_) and partially saturated magnetizations (*M*_0_′) were calculated as described in Reference 18. *T*_1_^app^ was calculated from

(1) $$T_{1}^{\text{app}}=-\frac{T_{R}}{\ln\left[\frac{\sin\;\alpha \;-\;R^{\prime }\cdot \sin\;\beta }{\cos\;\beta ▪\sin\;\alpha \;-\;R^{\prime }\cdot \cos\;\alpha \cdot \sin\;\beta }\right]}\text{,}$$

where *R*′ = *M*′(*α*)/*M*′(*β*) is the ratio of the partially saturated signals acquired with the two FAs, *α* and *β*.

Pseudo‐first‐order rate constants of the ATP synthesis reaction (*k*_ATP_) and the CK forward reaction (*k*_CK_) were then computed using

(2) $$k=\frac{1}{T_{1}^{\text{app}}}\left(1-\frac{M_{0}^{\prime }}{M_{0}}\right)\text{.}$$

**Figure 3:**
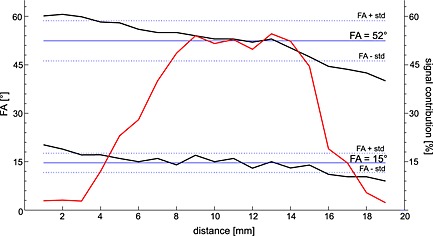
Representation of the local FA values, inside the localization map (upper black line represents FA = 52° and lower black line is FA = 15°) and corresponding signal intensity map (red line) along the slices.

For the absolute quantification of metabolite concentrations, a cellular ATP concentration of 8.2 mM was assumed 27. The corresponding forward metabolic fluxes (*F*_ATP_, *F*_CK_) were the products of *k*_ATP_ and *k*_CK_ and the concentrations of P_i_ and PCr, respectively 21.The potential error in the calculated *T*_1_^app^ (Equation [1]) and *k* (Equation [2]), based on the *B*_1_ variability, was calculated from the acquired *B*_1_ maps weighted by signal intensity. The spatial distribution of the actual *T*_1_^app^ was calculated inside the localization slab, using local FA values (Fig. 3, black lines). The local *T*_1_^app^ values were then weighted by the corresponding signal intensity (Fig. 3, red line). From these calculations we obtain the actual *T*_1_^app^, which was found to be very similar to the *T*_1_^app^ calculated using the nominally set FA 15° and 52°. The error in the rate constant *k* was calculated using the same approach.

A comparison between two methods (FAST and standard ST) was made using a Bland–Altman plot, which is used to evaluate the average discrepancy between methods and the limits of agreement 29. The limits of agreement were defined as the mean bias ± 1.96 times the standard deviation (SD). The repeatability of the FAST measurements was assessed using coefficients of variation (CVs), which are defined as the ratio of the SD to the mean value. Two‐tailed paired Student *t* tests were used to determine the significance of differences between the exchange rate constants and metabolic fluxes determined from standard ST and FAST measurements. Similarly, the comparison of *k*_ATP_/*k*_CK_ and *F*_ATP_/*F*_CK_ measured at rest and during exercise was also performed using paired *t* tests. A *p* value less than 0.05 was considered statistically significant.

## Results

The measured excitation frequency profiles of BIR4 pulses (i.e. sech/tanh and cos/sin) were insufficient, since adiabatic conditions were not achieved due to power restrictions and SAR limitations. The profiles of the 15° and 52° sinc excitation pulses (duration 600 µs) are depicted in Fig. 2b. Based on these results, sinc pulses were selected for excitation in further experiments.

*B*_1_ maps acquired *in vivo* showing the spatial distribution of the FAs using the selected sinc excitation pulse with our surface coil at 7 T are depicted in Fig. 2c (52°) and Fig. 2d (15°). Although the maps showed inhomogeneous distribution of FA over the total sensitivity volume of the coil, the homogeneity can be considered sufficient (CV inside the slab parallel to the coil was under 7% for FA = 15° and under 5% for FA = 52°) in a region close to the surface coil.

Therefore, to ensure accurate FA adjustments, a slice selective excitation, i.e. a DRESS sequence, was used 24, which was shown to provide accurate localization at 7 T 27. The mean FAs inside the localization slab measured *in vivo* were 51.4° ± 2.1° (Fig. 2c) and 16.4° ± 0.9° (Fig. 2d), for the nominally set excitation FAs of 52° and 15°, respectively. The off‐resonance behavior of the sinc pulse remained stable in the range ±10 ppm. The evaluated error in calculated *T*_1_^app^ based on FA inaccuracy was lower than 7%, and the error in *k* was under 6.5%.

**Figure 4:**
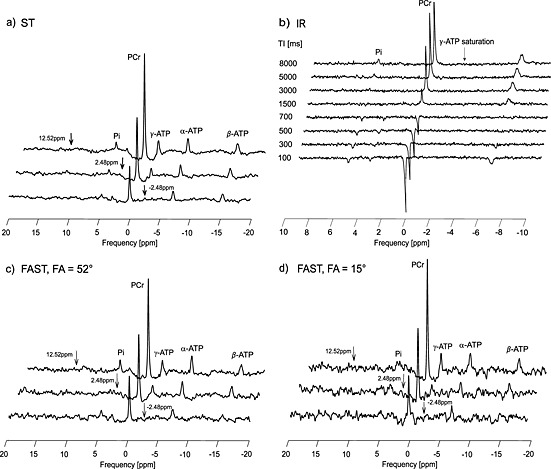
Representative spectra (acquired from the gastrocnemius medialis muscle) from the standard ST experiment, consisting of γ‐ATP and control saturations (a) and the IR experiment (b), and the FAST method measured with FA = 52° (c) and FA = 15° (d). Arrows depict the saturation frequency for each experiment; saturation of γ‐ATP at −2.48 ppm (bottom); control saturations for the PCr‐to‐ATP exchange rate at 2.48 ppm (middle), and for the P_i_‐to‐ATP exchange rate at 12.52 ppm (top).

Fig. 4 shows representative spectra from a standard ST experiment (Fig. 4a, b), spectra from the FAST method measured with FA = 52° (Fig. 4c), and spectra from the FAST method measured with FA = 15° (Fig. 4d). The frequency selective saturation pulse was set at three different offsets for each experiment: −2.48 ppm (bottom); 2.48 ppm (middle); and 12.52 ppm (top). In the FAST experiment all the variables, *T*_1_^app^, *M*_0_′, and *M*_0_, necessary for the calculation of rate constant *k* were derived from partially saturated measurements using the dual‐angle method, applied twice in a total of four acquisitions, whereas in a standard ST method saturation, control, and IR experiments are needed.

Results for the DRESS‐localized standard ST and FAST measurements of the P_i_‐to‐ATP and PCr‐to‐ATP forward reaction rate constants and metabolic fluxes in a human gastrocnemius muscle are listed in Table 1. The values for *k*_ATP_ and *k*_CK_ measured by the FAST method (*k*_ATP_ = 0.11 ± 0.05 s^−1^ and *k*_CK_ = 0.26 ± 0.05 s^−1^) are comparable to those measured by the standard ST (*k*_ATP_ = 0.12 ± 0.03 s^−1^ and *k*_CK_ = 0.27 ± 0.02 s^−1^) (*p* = 0.08 and *p* = 0.44). The differences between FAST and standard ST in *F*_ATP_ and *F*_CK_ were also not significant (*p* = 0.18 and *p* = 0.3). The CVs, obtained from repeatability measurements, of PCr‐to‐ATP and P_i_‐to‐ATP exchange rate constants and metabolic fluxes by the DRESS‐localized FAST method at 7 T remained 10% or less and 20% or less (see Table 1), respectively. The metabolite concentrations and *T*_1_^app^ are also listed in Table 1.

**Table 1:** Calculated apparent *T*_1_ values, *k* constants and metabolite concentrations and fluxes of the PCr‐to‐ATP and P_i_‐to‐ATP reactions given as mean ± SD from all eight volunteers and CV from repeatability of the six volunteers. There were no significant differences in any of the calculated parameters measured by the DRESS‐localized FAST and standard ST

	PCr‐to‐ATP				P_i_‐to‐ATP			
	[PCr] (mM)	*T*_1_^app^_PCr_ (s)	*k*_CK_ (s^−1^)	*F*_CK_ (mM s^−1^)	[P_i_] (mM)	*T*_1_^app^_Pi_ (s)	*k*_ATP_ (s^−1^)	*F*_ATP_ (mM s^−1^)
Standard ST		1.58 ± 0.12	**0.27 ± 0.02**	8.86 ± 2.36		3.22 ± 0.67	**0.12 ± 0.03**	0.66 ± 0.29
FAST	32.52 ± 9.28	1.52 ± 0.09	**0.26 ± 0.05**	8.41 ± 2.38	5.48 ± 1.95	3.05 ± 0.61	**0.11 ± 0.05**	0.58 ± 0.28
CV of FAST		(4%)	**(9%)**	(7%)		(8%)	**(19%)**	(17%)

**Figure 5:**
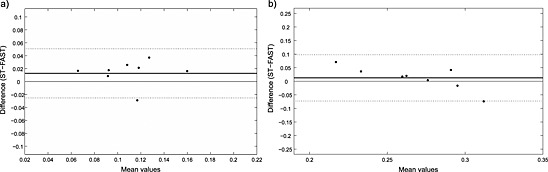
Plots of agreement between measurements of *k*_ATP_ (a) and *k_CK_* (b) by FAST and by standard ST at 7 T. The dotted lines represent upper and lower limits of agreement and the solid line represents the average of the difference.

Fig. 5 depicts the Bland–Altman agreement analysis for the two methods. The limits of agreement were narrow and the mean bias was close to zero for both the PCr‐to‐ATP and the P_i_‐to‐ATP exchange rate, as measured by the FAST and by standard ST.

**Figure 6:**
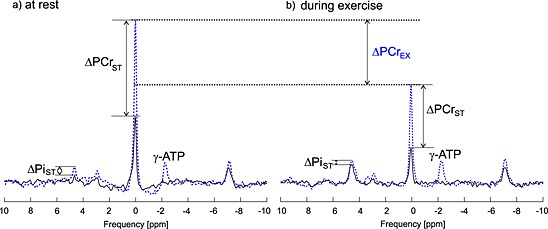
^31^P spectra showing FAST experiment, measured with FA = 52° (a) at rest and (b) during exercise with saturated γ‐ATP (black line) and with control saturation (blue line). Note that the spectra are scaled equally and the control experiment consists of two separate measurements, which were, for display purposes only, combined (the connection point is 2.5 ppm). The effect of exercise is visible as a depletion of PCr (highlighted by ΔPCr_EX_) and as an increase in P_i_ signal intensity.

Steady state spectra, measured with FA = 52° at rest (Fig. 6a) and during exercise (Fig. 6b) with saturated γ‐ATP (black line) and with control saturation (blue line) are compared in Fig. 6. ^31^P spectra show the effect of exercise on PCr and Pi signal intensities.

Results for the DRESS‐localized FAST measurements of the P_i_‐to‐ATP and PCr‐to‐ATP forward reaction rate constants and metabolic fluxes in a human gastrocnemius muscle, at rest and during exercise, are summarized in Table 2. During physical activity, the PCr‐to‐ATP metabolic flux decreased (from *F*_CK_ = 8.21 ± 1.15 mM s^−1^ at rest to *F*_CK_ = 3.86 ± 1.38 mM s^−1^ during exercise) and P_i_‐to‐ATP flux increased (from *F*_ATP_ = 0.43 ± 0.14 mM s^−1^ at rest to *F*_ATP_ = 0.74 ± 0.13 mM s^−1^ during exercise). The differences between the rates at rest and during exercise were significant for *k*_CK_ (*p* = 0.01), *k*_ATP_ (*p* = 0.009), and *F*_CK_ (*p* = 0.004), *F*_ATP_ (*p* = 0.03).

**Table 2:** Calculated values of metabolite concentrations, *F*, and *k* as mean ± SD obtained with FAST at rest, and with FAST during exercise in human gastrocnemius muscle of five volunteers at 7 T. Significant differences between the DRESS‐localized FAST measurements at rest and during exercise are indicated as *p < 0.05; ^#^ *p* < 0.01

	PCr‐to‐ATP				P_i_‐to‐ATP			
	[PCr] (mM)	*T*_1_^app^_PCr_ (s)	*k*_CK_ (s^−1^)	*F*_CK_ (mM s^−1^)	[P_i_] (mM)	*T*_1_^app^_Pi_ (s)	*k*_ATP_ (s^−1^)	*F*_ATP_ (mM s^−1^)
Rest FAST	30.62 ± 4.87	1.55 ± 0.27	0.27 ± 0.02	**8.21 ± 1.15**	3.53 ± 0.90	3.41 ± 0.38	0.12 ± 0.01	**0.43 ± 0.14**
Exercise FAST	21.32 ± 7.28	2.67 ± 0.44	0.19 ± 0.05*	**3.86 ± 1.38^#^**	15.82 ± 8.73	3.85 ± 0.37	0.05 ± 0.03**^#^**	0.74 ± 0.13*

## Discussion

In our study, we investigated the feasibility, repeatability, and possible increase in temporal resolution of the DRESS‐localized FAST technique measured on a 7 T MR system, to accelerate the measurement of both P*i*‐to‐ATP (ATP synthesis) and PCr‐to‐ATP (CK) reaction rates in the human gastrocnemius muscle. First, excitation pulse evaluation experiments were performed in the phantom. Second, the feasibility and repeatability of the DRESS‐localized FAST measurements were tested in volunteers at 7 T and compared with the reaction rates measured in the same subjects using the localized standard ST method; third, the feasibility of using the DRESS‐localized FAST at 7 T was tested for measurements of ATP metabolic fluxes in human gastrocnemius muscle during exercise.

Our data show that the measurements with the DRESS‐localized FAST method in the gastrocnemius muscle are feasible at 7 T, even without the use of adiabatic excitation pulses. The measured *k* values for PCr‐to‐ATP (*k*_CK_ = 0.26 ± 0.05 s^−1^) and P_i_‐to‐ATP (*k*_ATP_ = 0.11 ± 0.05 s^−1^) reactions are in good agreement with previously published results of localized experiments 12, 18, 30, 31. Bottomley *et al.* reported a *k*_CK_ for localized FAST of 0.29 ± 0.07 s^−1^. In their study, the localization was achieved by adding a 1D phase‐encoding gradient pulse after the excitation BIR4 pulse, and localized FAST data were acquired in 17–39 min (PCr‐to‐ATP exchange only) at 1.5 T 18. In a 3D‐TSE (turbo spin echo) imaging study of calf muscles at 7 T, Parasoglou *et al.* used continuous ST and IR to measure the PCr‐to‐ATP exchange rates of the gastrocnemius muscle (0.31 ± 0.05 s^−1^) in 60 min 31. The P_i_‐to‐ATP chemical exchange rate constant measured in the gastrocnemius muscle by Parasoglou *et al.* 12 in a different ^31^P‐MR imaging experiment at 7 T was 0.11 ± 0.04 s^−1^. The total acquisition time of the spectrally selective ST imaging experiment (P_i_ to ATP only), using a progressive saturation approach, was 45 min. Note that in both mentioned MRI‐based publications, the whole cross‐section of the calf was measured, thus providing exchange rate constants for several muscle groups in the same scan. Another recently published paper 30 reported a forward rate constant of the P_i_‐to‐ATP reaction of 0.09 ± 0.03 s^−1^ and a PCr‐to‐ATP of 0.27 ± 0.06 s^−1^ in the gastrocnemius muscle using a 1D‐ISIS (image‐selected *in vivo* spectroscopy)‐localized MRS ST method at 7 T. Their measurement time for both reactions in the gastrocnemius muscle was about 10 min.

The P_i_‐to‐ATP and PCr‐to‐ATP exchange rates measured in this study in the gastrocnemius medialis muscle by the FAST method were compared with the results of the standard ST method and visualized by a Bland–Altman plot. The agreement analysis of the two methods showed that the average of the difference value (bias) in *k*_CK_ and *k*_ATP_ values is very close to zero and the limits of agreement are narrow (i.e. within physiological differences). The CV was lower than 10% for *k*_CK_ and lower than 20% for *k*_ATP_. Thus, a DRESS‐localized FAST experiment can be performed at 7 T within 3.5 min with a sufficiently high repeatability, which suggests that the method can be used to measure dynamic changes, or when long scan times are impractical in patient studies, where the ST experiment is only a small part of the complex protocol.

To accelerate ST measurements and to overcome power requirements, bandwidth, and SAR limitations, Schär *et al.* 22 proposed a method called triple‐repetition‐time saturation transfer (TRiST). In their study, a dual‐repetition‐time sequence with frequency‐sweep‐cycled adiabatic half‐passage 90° pulses was used to avoid problems associated with BIR4 pulses at 3 T for *T*_1_^app^ measurements. The advantages of the TRiST method are, however, somewhat lessened by the inability to use a single *T*_R_ throughout the experiment, making it impractical for dynamic studies using a fixed trigger frequency (e.g. exercise–recovery experiments). Thus, the FAST method is a more suitable choice for such applications. Furthermore, signal localization to a specific muscle group, beneficial in exercise–recovery studies 32, is possible with our proposed approach, using a sinc excitation pulse, and cannot be implemented with adiabatic excitation without using time‐demanding phase encoding.

We have also shown that the DRESS‐localized FAST is feasible for the determination of metabolic fluxes during dynamic studies at 7 T. When measured during physical activity, the P_i_‐to‐ATP flux and P_i_ concentration were significantly higher compared with the measurements at rest.

The increment of P_i_‐to‐ATP flux during exercise found in our work (from 0.43 ± 0.14 mM s^−1^ to 0.74 ± 0.13 mM s^−1^) is in agreement with a recent dynamic study by Sleigh *et al.* 33. The aforementioned study reported the mean change of P_i_‐to‐ATP flux values measured at rest and during exercise depending on PCr recovery rate, using the standard ST technique. However, their experiment required extensive protocol, where the subject had to exercise twice, each time for over 12 min, which is not a clinically feasible approach. Another study assessing CK reaction in human skeletal muscle during exercise reported significant decrease in *F*_CK_ at the highest exercise level 34, which is in agreement with our results. The decrease in *F*_CK_ is attributed to metabolic compartmentalization and/or the reaction kinetics of a dead end complex stabilized by planar anions 34.

Both PCr‐to‐ATP and P_i_‐to‐ATP exchange rates measured during exercise were significantly lower than the exchange rates measured at rest. In the previously mentioned study on PCr‐to‐ATP kinetics during exercise 34, no differences were found in *k* values. However, there seems to be a tendency towards lower *k* once the PCr depletion starts to become significant. In the dynamic study by Sleigh *et al.* 33 no *k* values were given in the presentation, thus no direct comparison can be made. Nonetheless, decreased exchange rate constants during exercise are a little unexpected, and therefore further investigation of this phenomenon has to be performed.

The limitation of the FAST method is the dependence on an accurate FA adjustment. Bottomley *et al.* 18 used *B*_1_‐insensitive BIR4 excitation pulses at 1.5 T. Unfortunately, high *B*_1_^+^ requirements to reach adiabaticity would require long RF pulses with high *B*_1_^+^, thus exceeding SAR limitations at ultra‐high field. Therefore, the use of BIR4 excitation pulses at 7 T for our application was not feasible. As an alternative, we used a conventional excitation sinc pulse. The frequency pulse profile is very stable, but the ability to achieve homogeneous FA excitation is limited. The measured FA maps show sufficient homogeneity in a region parallel to the surface coil. Thus, for the localization, we used the slice selective localization scheme DRESS. However, the calf muscle anatomy does not allow the slab position parallel to the coil, which was fixed to the table/ergometer. Therefore, we used an oblique slab position trying to stay parallel to the coil as much as possible, but still in the gastrocnemius muscle. A more homogeneous *B*_1_^+^ coverage could be achieved using a volume coil 35, 36, an array of RF coils 37 or simply a larger surface coil 18. This would be especially beneficial for human subjects with large layers of subcutaneous fat, which acts as a pad or cushion between the muscle and the coil 35. Similarly, a shifted coil position, more parallel to the localization slab, could be used to improve the *B*_1_^+^ the homogeneity in the slab.

Nevertheless, the mean FAs inside our non‐parallel localization slab were 51.5° ± 2.1° and 16.4° ± 0.9°, for nominally set 52° and 15° excitation FAs. Furthermore, the calculated errors in *T*_1_^app^ and *k* for the measured *B*_1_^+^ variability were below 7% and below 6.5%, respectively.

In conclusion, we were able to show that the DRESS‐localized FAST technique for measurements of the human gastrocnemius muscle at ultra‐high field is feasible, highly repeatable, and provides reaction rates with 3.5 min temporal resolution. The results were comparable to those obtained via standard ST. In addition, we have shown that measurements of exchange rate constants and metabolic fluxes are feasible during steady state exercise using the DRESS‐localized FAST technique at 7 T.

Time‐resolved measurements should be possible with the DRESS‐localized FAST method and offer new opportunities for studies of human metabolism by ^31^P‐MRS, especially when a short acquisition time is critical.
